# Epidemiological analysis of coronary heart disease and its main risk factors: are their associations multiplicative, additive, or interactive?

**DOI:** 10.1080/07853890.2022.2078875

**Published:** 2022-05-23

**Authors:** Ari Voutilainen, Christina Brester, Mikko Kolehmainen, Tomi-Pekka Tuomainen

**Affiliations:** a Institute of Public Health and Clinical Nutrition, University of Eastern Finland, Kuopio, Finland; b Department of Environmental and Biological Sciences, University of Eastern Finland, Kuopio, Finland

**Keywords:** Additive, coronary heart disease, cohort study, incidence, multiplicative, interactive

## Abstract

**Objective:**

The purpose of this study was to discover how considering multiplicative, additive, and interactive effects modifies results of a prospective cohort study on coronary heart disease (CHD) incidence and its main risk factors.

**Material and methods:**

The Kuopio Ischaemic Heart Disease Risk Factor (KIHD) Study provided the study material, 2682 Eastern Finnish middle-aged men, followed since the 1980s. We applied multiplicative and additive survival models together with different statistical metrics and confidence intervals for risk ratios and risk differences to estimate the nature of associations.

**Results:**

The mean (SD) follow-up time among men who were free of CHD at baseline (*n* = 1958) was 21.4 (10.4) years, and 717 (37%) of them had the disease and 301 (15%) died for CHD before the end of follow-up. All tested non-modifiable and modifiable risk factors statistically significantly predicted CHD incidence. We detected three interactions: circulating low-density lipoprotein cholesterol (LDL-C) × age, obesity × age, and obesity × smoking of which LDL-C × age was the most evident one. High LDL-C increased the risk of CHD more among men younger than 50 [risk ratio (RR) 2.10] than those older than 50 (RR 1.22). LDL-C status was the only additive covariate. The additive effect of high LDL-C increased almost linearly up to 18 years and then reached a plateau. The simple multiplicative survival model stressed glycemic status as the strongest modifiable risk factor for developing CHD [hazard ratio (HR) for diabetes vs. normoglycemia was 2.69], whereas the model considering interactions and time dependence emphasised the role of LDL-C status (HR for high LDL-C vs. lower than borderline was 4.43). Age was the strongest non-modifiable predictor.

**Conclusions:**

Including covariate interactions and time dependence in survival models potentially refine results of epidemiological analyses and ease to define the order of importance across CHD risk factors.

**KEY MESSAGES**
- Including covariate interactions and time dependence in survival models potentially refine results of epidemiological analyses on coronary heart disease.
- Including covariate interactions and time dependence in survival models potentially ease to define the order of importance across coronary heart disease risk factors.

## Introduction

Coronary heart disease (CHD) is one of the most common chronic illnesses in the world and its main risk factors are well-known [1]. These risk factors are male gender, old age, ethnic background, family history of CHD, diabetes, smoking, obesity, dyslipidemia, hypertension, and physical inactivity of which the first four are non-modifiable and the latter six are modifiable [2,3]. Out of many unorthodox predictors of CHD, for example, proteinuria and inflammatory biomarkers have shown strong associations with CHD incidence, but their causal roles in the development of CHD are typically uncertain [4–7].

Traditionally, most epidemiological studies and treatment algorithms have considered the CHD risk factors as independent predictors [8], which may distort conclusions due to unidentified interactions. This is somewhat surprising as studies addressing the interactions across CHD risk factors have emphasised their importance and revealed associations of modifiable risk factors particularly with the family history and genetic risk [9–13], but also across the modifiable risk factors, for example, between dyslipidemia and smoking, dyslipidemia and hypertension, dyslipidemia and obesity, and diabetes and hypertension [11,14,15].

Moreover, epidemiological studies have typically not investigated the type of the relationship between CHD and its risk factors, multiplicative or additive, before choosing statistical methods and simply applied multiplicative models, such as the Cox regression analysis. With respect to CHD, fortunately, multiplicative models are probably the correct choice for many studies, as the effects of coexisting CHD risk factors as such appear to be multiplicative rather than additive, although also additive effects evidently exist [8,14,16,17]. In general, on the other hand, additive models should be more appropriate in determining epidemiological relationships [18].

The aim of this prospective study was to discover the combination of multiplicative, additive, and interactive effects that best recognise the main risk factors for CHD incidence and their order of importance in a cohort of middle-aged men followed since the 1980s.

## Material and methods

### Material

The ethical committee of the Kuopio University approved the KIHD Study on December 1, 1983. The KIHD study plan was published in 1988 [19] and the actualised study protocol in separate papers in the late 1980s and early 1990s. Salonen et al. [20] provides a comprehensive presentation concerning variables applied in the present study. Briefly, the primary KIHD study sample consisted of 3235 middle-aged men living in Eastern Finland in the city of Kuopio and its surrounding rural areas. Of those men, 2682 were willing to participate to baseline examinations and interviews between March 1984 and December 1989. All study participants gave written informed consent.

To enable the identification of study participants who were not free of CHD at baseline, KIHD examinations included a maximal symptom-limited exercise tolerance test carried out at the Kuopio Research Institute of Exercise Medicine [21]. Bicycle ergometers with a linear (Medical Fitness Equipment 400 L, Mearn, the Netherlands) or a step-by-step (Tunturi EL 400, Turku, Finland) increase in the workload by 20 W per minute served as devices for the assessment of physical work. Measurements of oxygen uptake were based on a breath-by-breath method (MGC 2001, Medical Graphics, St. Paul, MN) or a mixing-chamber method (Mijnhardt Oxycon 4, Odijk, the Netherlands). The test procedure consisted standard 12-lead ECG recordings (Kone, Turku, Finland) before, during, and after the ergometer test. The before recordings corresponded a resting ECG. Moreover, KIHD questionnaires included the following CHD-related questions: (a) has your physician told you that you have had a myocardial infarction, (b) has your physician told you that you suffer from angina pectoris, (c) have you used medicines for angina pectoris during the past 7 days, (d) do you use sublingual nitroglycerine once a week or more frequently, and (e) have you undergone a coronary bypass operation? Based on results of the exercise test and ECG recordings and answers to questionnaire items we defined study participants having CHD at baseline as follows: unable to complete the ergometer test due to angina pectoris-type chest pain, or Q waves on the ECG indicating a myocardial infarction, or horizontal or downsloping ST depression ≥1 mm in aVF or V5 leads, or answering “yes” to at least one of the questions a − e. As certain inaccuracies relate to exercise stress testing in general [22], in this study, we carried out statistical analyses and reported their results also concerning the dataset without exclusions.

Before giving blood samples between 8 and 10 a.m. study participants abstained from alcohol for 3 days, smoking for 12 h, and eating for 12 h. After a rest of 30 min, a research nurse drew blood with Terumo Venoject VT-100PZ vacuum (Terumo Corp., Tokyo, Japan) without using a tourniquet.

### Endpoints

Study endpoints were i) CHD diagnosed during hospitalisations or specialised medical care visits, ii) death, and iii) the end of follow-up on 31 December 2019. CHD referred to ICD 10 codes I20 − I25. The KIHD Study receives ICD 10 codes *via* annual linkages to the Care Register for Health Care provided by the Finnish Institute for Health and Welfare (License THL/93/5.05.00/2013 valid until 31 December 2022) and verifies deaths by annual linkages to the Causes of Death Register provided by the Statistics Finland (License TK-53-1770-16 valid until 31 December 2026).

### Covariates

Family history of CHD based on the following questionnaire items: (a) has your biological mother CHD, (b) has your biological father CHD, and (c) have your biological siblings CHD? This covariate combines effects of heredity and familial environment on the risk of CHD and, consequently, it does not represent genetic factors as such or effects of adulthood environment.

Age referred to four categories, 42, 48, 54, and 60 years, reflecting the primary KIHD recruitment.

The assessment of the absence and presence of borderline diabetes and diabetes at baseline based on a fasting blood glucose (FBG) level measured by a glucose dehydrogenase method (Merck, Darmstadt, Federal Republic of Germany) together with the following questionnaire items: (a) do you have diet-controlled diabetes, (b) do you use oral diabetes medications, and (c) do you use insulin? For statistical analyses we categorised study participants into three groups as follows: FBG <5.6 mmol/L indicating no diabetes, FBG 5.6 − 6.9 mmol/L and/or diet for controlling diabetes, and FBG ≥7.0 mmol/L and/or glucose-lowering medication indicating diabetes [23,24]. We classified men with diet-treated diabetes and men taking diabetes medicines into different groups as, based on preliminary analyses, the risk of CHD was evidently higher among the latter.

Smoking status based on a self-administered questionnaire of which items dealt with the frequency and duration of regular smoking as well as the types of tobacco products consumed. We classified the study participant as a smoker if he had ever smoked regularly and a current smoker if he had smoked regularly within the past 30 days.

KIHD baseline examinations included height and weight measurements. We calculated the Body Mass Index (BMI) for each study participants by dividing the weight in kilograms by the square of height in metres and categorised study participants as follows: BMI <25.0 kg/m^2^ indicating healthy weight, BMI 25.0–29.9 kg/m^2^ indicating overweight, and BMI ≥30.0 kg/m^2^ indicating obesity [25].

In this study, we used the fasting serum low-density lipoprotein cholesterol (S-LDL-C) concentration instead of the fasting serum total cholesterol (STC) concentration to detect high cholesterol levels. In our previous paper, we had used STC [26], but preliminary analyses of this study revealed that S-LDL-C could provide even stronger associations with CHD. Based on S-LDL-C concentration we categorised study participants as follows: S-LDL-C < 3.4 mmol/L indicating below borderline concentrations, S-LDL-C 3.4 − 4.1 mmol/L indicating borderline high concentrations, and S-LDL-C > 4.1 mmol/L or cholesterol-lowering medication indicating high concentrations. These cut-off points do not strictly follow clinical guidelines or research findings concerning desirable S-LDL-C levels [27] because, in the KIHD cohort, the proportion of men with the desirable S-LDL-C concentration for individuals at low risk (<3.0 mmol/L) is low (14.7%) whereas men having dyslipidemia are overrepresented. Therefore, we combined optimal and near optimal S-LDL-C groups into a below borderline group to achieve as balanced 3-group distribution as possible for statistical analyses. To some extent, the desirable S-LDL-C levels are always only directional as, by and large, the lower the circulating LDL-C concentration, the lower the risk of cardiovascular diseases [28]. Salonen et al. [29] describe the KIHD lipid analyses in detail. Briefly, the main fractions, very low-density lipoprotein, S-LDL-C, and high-density lipoprotein (HDL) referred to the respective serum fractions: the top fraction, a computational difference between the bottom and HDL fractions, and the supernatant after precipitation of the bottom fraction.

KIHD baseline examinations included several blood pressure measurements. In this study, we refer to the mean of three measurements in supine, two in sitting, and one in a standing position with a random-zero mercury sphygmomanometer after a supine rest of five minutes. Based on systolic (SBP) and diastolic blood pressure (DBP) values we categorised study participants as follows: SBP <120 and DBP <80 mmHg indicating no hypertension, SBP 120 − 139 or DBP 80 − 89 mmHg indicating borderline hypertension, and SBP >139 or DBP >89 mmHg or blood pressure medication indicating hypertension. These cut-off points are compromises across clinical guidelines, research findings, and distributions of SBP and DBP values in the KIHD cohort [30,31]. Mainly, they follow criteria applied in the Framingham Heart Study that resembles, in many ways, the KIHD Study [32].

We did not use ethnic background and gender as covariates because all study participants were White Finnish males. We also excluded measures of physical activity since the proportion of physically inactive men in the KIHD cohort is practically zero and, consequently, it is impossible to estimate the impact of sedentary lifestyle on the risk of CHD. Moreover, based on our previous analyses physical activity levels very poorly predict the risk of CHD in this cohort [26]. Other studies also have found the contradictory nature of physical activity, as in some study populations physical activity, not inactivity, is associated with an increased risk of CHD [9].

### Statistical analyses

To detect interactions, we computed risk ratios (RR) and risk differences (RD) with 95% confidence intervals (CI) for each pair of covariates. In RD, CIs refer to the Wilson intervals [33]. We also fitted a binomial regression model as a part of generalised linear models to each pairwise interaction. Basically, non-overlapping confidence intervals indicate statistically significant interactions, RRs regarding multiplicative interactions and RDs regarding additive interactions. Moreover, we used the interaction contrast ratio (ICR), with 95% CIs based on the variance recovery method [34], to verify additive interactions. ICR expresses the additional risk due to the interaction and it is also known as RERI, the relative risk for interaction [35–37]. If the additive interaction is present, ICR differs from zero. We also calculated the attributable fraction and the synergy index [35,37] as a part of a preliminary analysis, but them did not affect conclusions based on ICR.

To simplify the interpretation of interactions we dichotomised the covariates as follows: Family history (no close relatives with CHD vs. close relative(s) with CHD), age (<50 vs. >50), glycemic status (FBG <5.6 mmol/L and no glucose-lowering medication or diet vs. FBG ≥5.6 mmol/L or glucose-lowering medication or diet), smoking status (never smoked vs. former or current smoker), weight (BMI <30 kg/m^2^ vs. BMI ≥30 kg/m^2^), cholesterol status (S-LDL-C ≤ 4.1 mmol/L and no cholesterol-lowering medication vs. S-LDL-C > 4.1 mmol/L or cholesterol-lowering medication), and blood pressure status (SBP ≤139 mmHg, DBP ≤89 mmHg, and no blood pressure medication vs. SBP >139 mmHg, or DBP >89 mmHg, or blood pressure medication).

Regarding multiplicativity and additivity we, first, performed a conventional Cox proportional hazards model that assumes multiplicative associations [38]. Precisely, the proportional hazard assumption denotes that covariate coefficients do not change over time, i.e. *β*(*t*) = *c* [39]. In the Cox survival model, the CHD endpoint served as the dependent variable and all covariates served as independent variables. We used the covariates as categorical variables and investigated the Schoenfeld residuals to test the proportional hazards assumption [40]. Based on our previous study the type of covariate, categorical or continuous, has probably no statistically significant effect on Cox model results [26] but including continuous covariates in the model requires the assessment of nonlinearity by means of martingale residuals [41]. Second, we added statistically significant covariate interactions to the Cox model. Third, we performed the Cox-Aalen model with a proportionality test based on martingale residuals [42,43]. The Cox-Aalen model allows covariates to result in either multiplicative or additive effects [43]. Fourth, to estimate effects of additive covariates we executed the Aalen’s additive regression model [44,45]. As the KIHD study utilises the IBM^®^ SPSS^®^ software platform, we also applied the SPSS built-in time variables to estimate effects of additive covariates. When covariates are time-dependent, another option is to divide the follow-up period into shorter covariate-specific time segments. In this study, however, we did not test this method, since it may lead to the use of even several different time segments if the number of time-dependent covariates is high. However, in case of few covariates the method is worth exploring [39].

To better illustrate the possible additive nature of CHD risk factors we created a severity grading from 0 to 15. Grade 0 referred to the following baseline characteristics: No close relatives with CHD, age 42, FBG <5.6 mmol/L and no glucose-lowering medication or diet, never smoked, BMI <25 kg/m^2^, S-LDL-C < 3.4 mmol/L and no cholesterol-lowering medication, and SBP <120 mmHg, DBP <80 mmHg, and no blood pressure medication. Correspondingly, Grade 15 referred to the most severe risk profile with respect to CHD risk factors at baseline (Table 1). We do not suggest the grading as an alternative for well-established CHD risk scores, for example, but a way to concretise cohort-specific effects of CHD risk factors. In the grading, we applied the same severity classes as in other analyses to maintain coherence across results.

**Table 1.** Distribution of study participants by the main CHD risk factors at baseline.

Risk factor	Severity	Description	*n* (*N*)
Age	0	42 years	294 (334)
	1	48 years	274 (358)
	2	54 years	1148 (1592)
	3	60 years	242 (398)
Family history	0	No close relatives with CHD	1061 (1351)
	1	One close relative with CHD	638 (898)
	2	Two or more close relatives with CHD	244 (413)
			missing 15 (20)
Diabetes	0	FBG <5.6 mmol/L	1800 (2425)
	1	FBG 5.6 − 6.9 mmol/L or diagnosis and diet	102 (159)
	2	FBG >6.9 mmol/L or medication	56 (98)
Smoking	0	Never	672 (861)
	1	Previously	675 (955)
	2	Currently	611 (866)
Obesity	0	BMI <25 kg/m^2^	622 (843)
	1	BMI 25 − 29.9 kg/m^2^	993 (1351)
	2	BMI ≥30 kg/m^2^	334 (476)
			missing 9 (12)
Cholesterol	0	S-LDL <3.4 mmol/L	539 (705)
	1	S-LDL 3.4 − 4.1 mmol/L	568 (772)
	2	S-LDL >4.1 mmol/L or medication	816 (1156)
			missing 35 (49)
Hypertension	0	SBP <120 and DBP <80 mmHg	205 (257)
	1	SBP 120 − 139 or DBP 80 − 89 mmHg	645 (799)
	2	SBP >139 or DBP >89 mmHg or medication	1098 (1614)
			missing 10 (12)

*Notes*. *n* refers to 1958 men free of coronary heart disease (CHD) at baseline, and N refers to the entire cohort of 2682 men. FBG: fasting blood glucose; BMI: body mass index; S-LDL: serum low-density lipoprotein; SBP: systolic blood pressure; DBP: diastolic blood pressure.

R 4.0.2 with the R packages “interactionR,” “survival,” and “timereg” served as a statistical platform [43,46–50].

## Results

### Baseline characteristics

All study participants were men, and their respective mean (SD) age, FBG concentration, BMI, S-LDL-C concentration, SBP, and DBP were 53.1 (5.1) years, 4.8 (1.2) mmol/L, 26.9 (3.6) kg/m^2^, 4.0 (1.0) mmol/L, 134.2 (17.1) mmHg, and 88.7 (10.5) mmHg. Among those, who had ever smoked (*n* = 1821) the mean (SD) pack-year was 16.2 (18.9). Based on baseline examinations and interviews 724 men had CHD. Table 1 presents distributions of study participants by CHD risk factors and risk factor severity gradings.

### Endpoints

Of men who were free of CHD at baseline (*n* = 1958), 717 (37%) had the disease and 301 (15%) died for CHD before the end of follow-up. All in all (*n* = 2682), 1777 (66%) men died, 541 (20%) men died for CHD, 905 (34%) men were alive, and 737 (27%) men were alive and free of CHD at the end of follow-up. The mean (SD) follow-up time was 21.4 (10.4) years among men who were free of CHD at baseline, 14.7 (10.6) years among men who had CHD at baseline (*n* = 724), and 19.6 (10.8) years among all men. The respective risks of all-cause (and CHD) mortality were 0.62 (0.15), 0.77 (0.33), and 0.66 (0.20).

### Interactions

**Figure 1.**
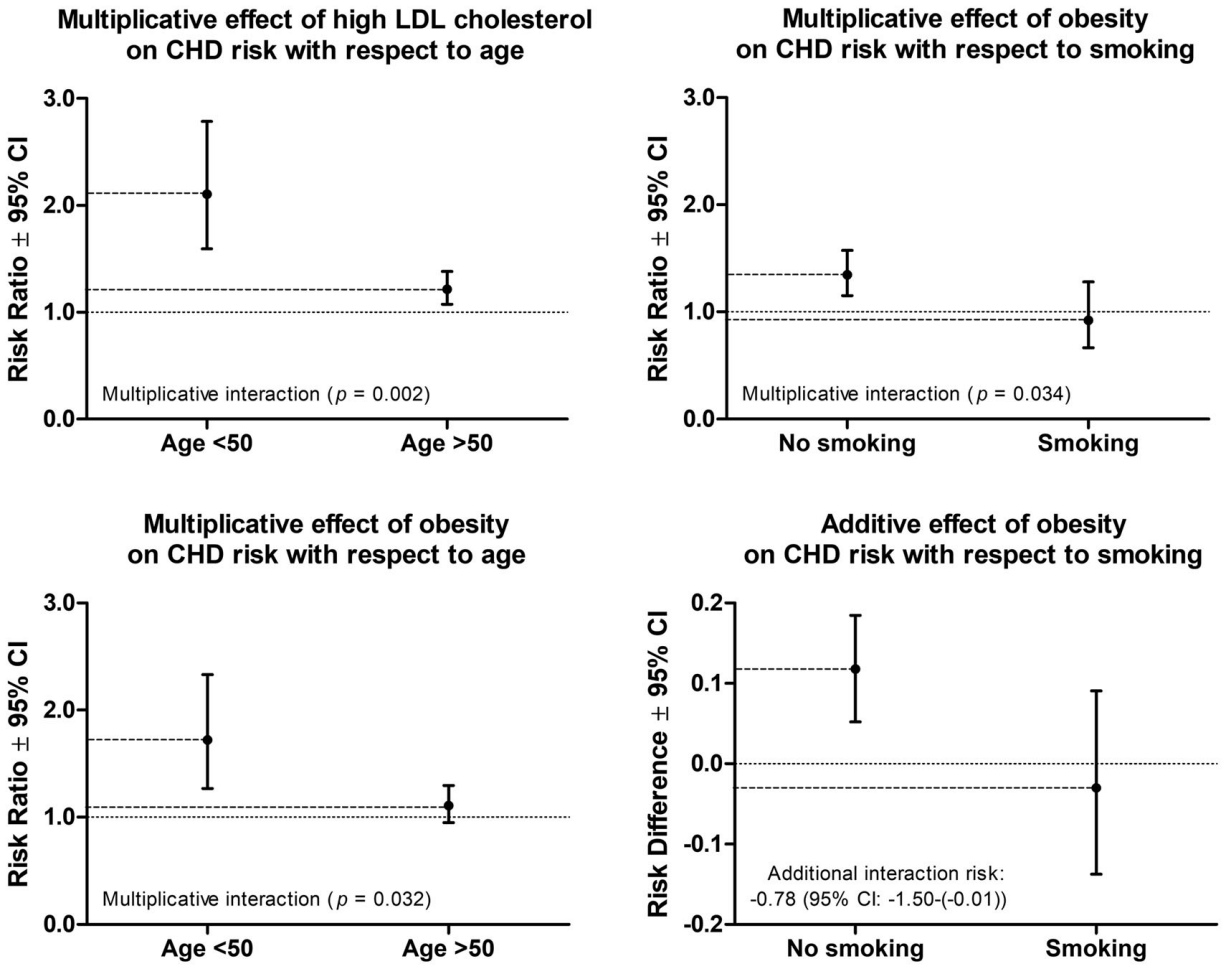
Statistically significant interactions across the main coronary heart disease (CHD) risk factors.

We detected three multiplicative and one additive interactions across the covariates (Figure 1, Supplementary table 1). High S-LDL-C and obesity increased the risk of CHD more among men younger than 50 (RR for high S-LDL-C: 2.10, RR for obesity: 1.72) than those older than 50 (RR for high S-LDL-C: 1.22, RR for obesity: 1.11), and obesity increased the risk of CHD particularly among non-smokers (RR in non-smokers: 1.35, RR in smokers: 0.92). The interaction between weight and smoking statuses was significant both at multiplicative and additive scales.

### Multiplicative and additive effects

Most covariates were multiplicative. In a basic multiplicative survival model, glycemic status related to the highest hazard ratio (HR). After considering covariate interactions and time dependence, the model emphasised age, weight status, and glycemic status as the strongest multiplicative covariates with the respective category-to-category HRs of 1.94, 1.68, and 1.63 (Table 2). The HR (95% CI) of age 60 compared to age 42 was 5.69 (2.22 − 14.58), that of obesity compared to normal weight was 1.72 (0.78 − 3.81), and that of diabetes compared to no diabetes was 2.75 (1.89 − 4.00). Improvements in the model fit from the basic model to the model with interactions and further to the model with interactions and time dependence were statistically only modest. In the first step, the −2 times the log of the likelihood (-2LL) decreased from 16,055 to 16,034 with the *p*-value of .183 and in the second step, -2LL decreased from 16,034 to 16,028 with the *p*-value of .461.

**Table 2.** CHD risk factor specific category-to-category hazard ratios (95% CI) together with *p*-values without and with covariate interactions and time dependence.

Risk factor	Model 1	Model 2	Model 3
Age of 42 years	1.48 (1.35 − 1.63)	1.91 (1.52 − 2.40)	1.94 (1.54 − 2.44)
Age of 48 years	*p* < .001	*p* < .001	*p* < .001
Age of 54 years		× Weight = 0.152	× Weight = 0.156
Age of 60 years		× S-LDL-C = 0.036	× S-LDL-C = 0.023
No family history	1.30 (1.17 − 1.44)	1.29 (1.16 − 1.43)	1.29 (1.16 − 1.43)
One with CHD	*p* < .001	*p* < .001	*p* < .001
Two or more with CHD			
No diabetes	1.64 (1.39 − 1.92)	1.63 (1.38 − 1.91)	1.63 (1.38 − 1.91)
Borderline or diet-treated	*p* < .001	*p* < .001	*p* < .001
Diabetes			
Never smoking	1.31 (1.19 − 1.45)	1.44 (1.23 − 1.69)	1.45 (1.23 − 1.70)
Previous smoker	*p* < .001	*p* < .001	*p* < .001
Current smoker		× Weight = 0.142	× Weight = 0.132
Normal weight	1.26 (1.13 − 1.42)	1.68 (1.23 − 2.29)	1.68 (1.23 − 2.30)
Overweight	*p* < .001	*p* = .001	*p* = .001
Obesity		× Age = 0.152	× Age 0.156
		× Smoking = 0.142	× Smoking = 0.132
Below borderline S-LDL-C	1.30 (1.18 − 1.42)	1.64 (1.29 − 2.08)	1.95 (1.43 − 2.67)
Borderline	*p* < .001	*p* < .001	*p* < .001
High S-LDL-C		× Age = 0.036	× Age = 0.023
			× TIME = 0.073
No hypertension	1.31 (1.16 − 1.49)	1.32 (1.16 − 1.49)	1.32 (1.17 − 1.50)
Borderline	*p* < .001	*p* < .001	*p* < .001
Hypertension			

*Notes*. Study participants were free of coronary heart disease (CHD) at baseline (*n* = 1958). Model 1: Basic multiplicative survival model. Model 2: With covariate interactions. Model 3: With covariate interactions and time dependence. S-LDL-C: Serum low-density lipoprotein cholesterol.

**Figure 2.**
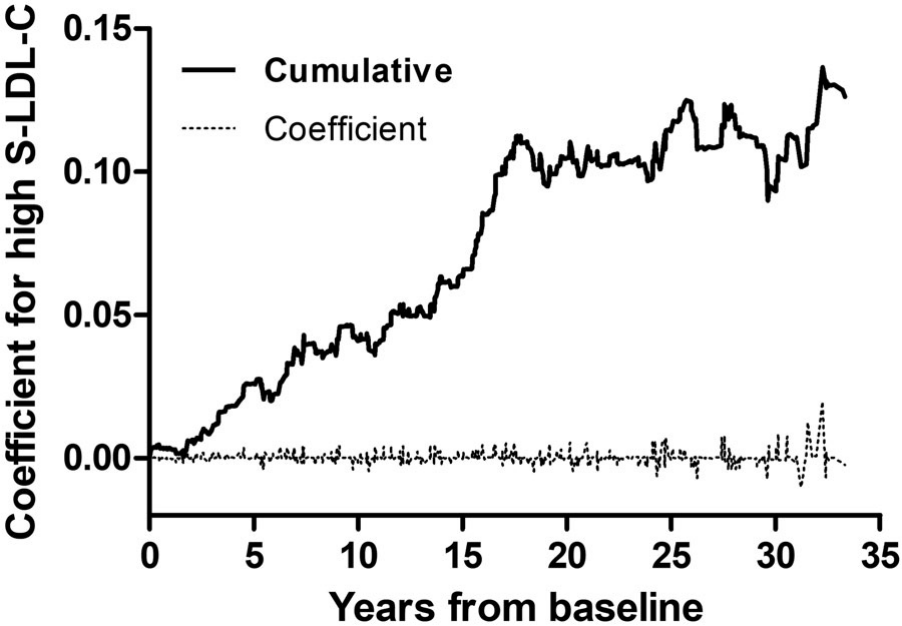
Additive effect of high serum low-density lipoprotein cholesterol (S-LDL-C) concentrations on the risk of coronary heart disease.

The only additive covariate was S-LDL-C status. Schoenfeld residuals indicated no violations of the proportional hazards assumption whereas the proportionality test pointed out S-LDL-C status. The *p*-value was = .033 when the model included covariate interactions and .043 when it did not. The additive effect of borderline high S-LDL-C compared to below borderline S-LDL-C and that of high S-LDL-C compared to borderline high S-LDL-C suggested two extra CHD cases per 1000 men during 10 years of follow-up, as the mean coefficient was 1.84 × 10^−4^. The cumulative coefficient for S-LDL-C status increased almost linearly up to 18 years with the slope of c. 0.006 and then reached a plateau (Figure 2). When comparing high S-LDL-C to the combined group of borderline high and below borderline S-LDL-C, the additive effect suggested 2.5 extra CHD cases per 1000 men during 10 years of follow-up with the mean coefficient of 2.51 × 10^−4^.

**Figure 3.**
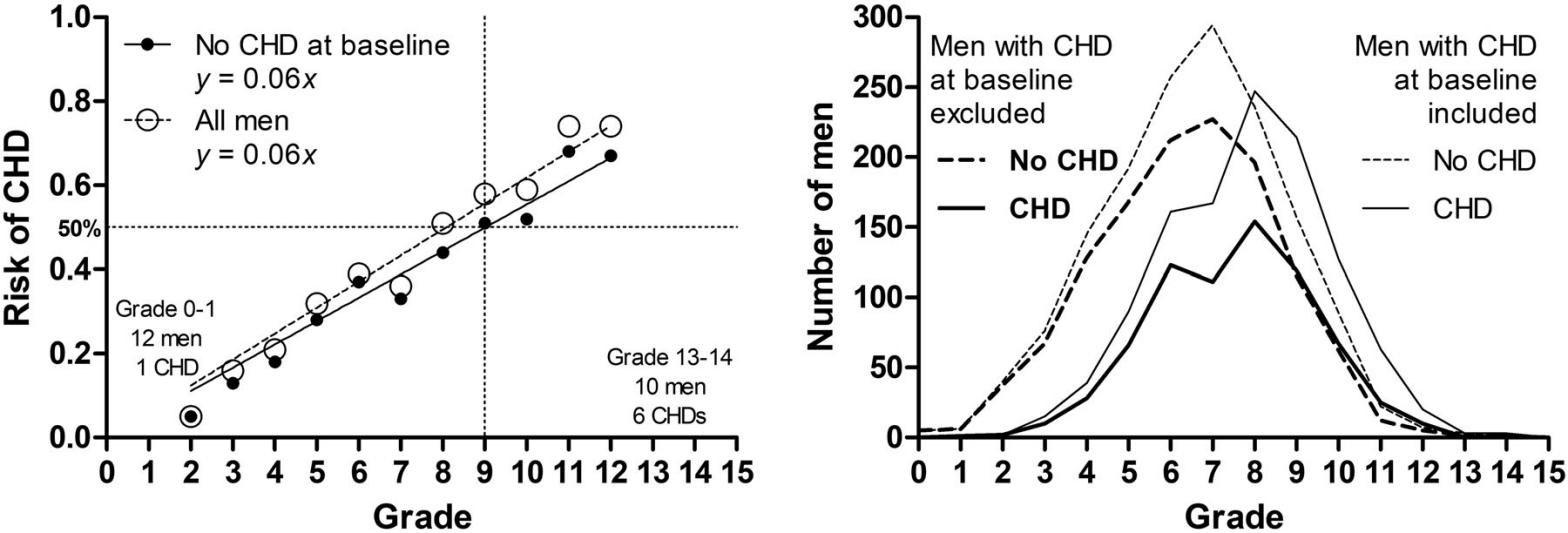
Relationship between the risk of coronary heart disease (CHD) and the severity grading of CHD risk factors.

Concerning the severity grading of CHD risk factors, a one-point increase in the severity score increased the absolute risk of CHD by 6% regardless of whether men with CHD at baseline were included or excluded (Figure 3). From Grades 2 to 12, the relationship was linear with the *p*-value of <.0001, and it explained 97% of the variation in the absolute CHD risk. Between Grades 12 and 2, the respective RD (95% CI) and RR (95% CI) were 0.62 (−0.45 − 0.85) and 13 (3.22 − 52.54). The CIs were wide due to low number of men belonging to these grade categories, 15 and 39, respectively. Computationally, the 6% point-to-point increase in the risk corresponds to the RD of 0.60 and RR of 6.00, when the interval is 10 points. If including the uttermost grades (0, 1, 13, and 14) and men with CHD at baseline, the simple linear regression still explained 89% of the variation. Grade distributions followed a normal distribution, and no men reached the maximum score of 15 (Figure 3).

## Discussion

Based on our findings considering covariate interactions and time dependence in survival modelling may refine results and ease to define the order of importance across the main CHD risk factors. Previous simulations and real data demonstrations have proposed that extending conventional Cox survival models improves model fit when predicting CHD by means of its risk factors [16,51]. Additive models also appear to emphasise partly different CHD risk factors than multiplicative models [16,17] and replacing both additive and multiplicative models with other techniques, such as survival trees and Fuzzy Logic-based models, highlights yet more risk factors [52,53]. Traditionally, studies have identified dyslipidemia as a pivotal CHD risk factor [8]. Comparisons across studies with respect to the order of significance among risk factors, however, are difficult because studies have applied different risk factor combinations and within-covariate variations greatly differ across studies. Among the main modifiable CHD risk factors, studies have pointed out at least unfavourable glycemic status [9,10] and smoking [6,51,52] as the strongest one, and survival tree analyses have suggested slightly different combinations of CHD risk factors for different age groups and genders [52].

Our study suggested high S-LDL-C as the strongest modifiable predictor of CHD in middle-aged men, which is in accordance with the traditional opinion [8]. At the same time, our study revealed the interaction between high S-LDL-C and age, the strongest non-modifiable predictor, as well as the time dependence of high S-LDL-C, as the relative significance of high S-LDL-C diminished together with increasing age and follow-up time. This time dependent interaction also is in accordance with earlier findings [54]. However, without considering both interactions and time dependence in the statistical model this study would have stressed other covariates, such as glycemic status, and omitted the importance of high S-LDL-C.

The associations of CHD with its main risk factors in the KIHD cohort appeared to be additive in that sense that each one-point increase in the risk factor severity grading increased the absolute risk of CHD just about equally and irrespective of the risk factor type. For example, becoming 6 years older, becoming overweight, and developing a precondition equally increase the risk of CHD i.e. by 6% over the next three decades. This finding for its part supports the traditional opinion of CHD as a multifactorial disease without any single dominant risk factor [8].

The additive effect of obesity on the CHD risk in the presence of smoking was negative i.e. the absolute risk of CHD was slightly lower among obese smokers (0.35) than among normal or overweight smokers (0.38). This effect for its part reflects the fact that smoking per se increases the risk of all-cause mortality, even more than obesity does [55]. In the KIHD cohort, the absolute risk of all-cause and non-CHD mortalities are highest specifically among non-obese smokers (0.78 and 0.61), evidently higher than among obese smokers (0.73 and 0.50). Correspondingly, multiplicative interactions of age with high S-LDL-C and obesity suggested the significance of high S-LDL-C and obesity among men younger than 50. A reason for this somewhat surprising age-obesity relationship can partly relate to the duration of obesity, as the KIHD study participants who were obese and younger than 50 at KIHD baseline had the highest mean (95% CI) BMI in young adulthood (23.3, 22.9 − 23.8), statistically significantly (*p* = .001) higher than that of obese study participants who were older than 50 at KIHD baseline (22.5, 22.3 − 22.7). In other words, the KIHD study participants who were obese and younger than 50 at KIHD baseline could have exposed to obesity longer than the KIHD study participants who were obese but older than 50 at KIHD baseline. Irrespective of the direction of the interaction, however, the interaction should be considered in statistical models.

All in all, considering how long and intensively studies have underlined the importance of combining multiplicative, additive, and interactive effects in general and in the epidemiology of CHD [8–18], it is astonishing that covariate interactions and their time dependencies do not belong to the routine statistical procedures of epidemiological studies on CHD. One reason for the inequality between multiplicative and additive survival models may relate to the interpretation of their results. Results of multiplicative models can be understood as risk ratios but results of additive models should be at least partly reported as risk differences and additional cases, which, however, does not denote additive models are difficult to interpret per se [56].

## Strengths and limitations

The present study is a longitudinal study with an exceptionally long follow-up period, whereas a whole range of previous findings concerning the nature of associations across CHD and its main risk factors originate from cross-sectional and case-control studies. This study also included information from all 10 main non-modifiable and modifiable CHD risk factors, whereas previous studies have typically included only some of them. On the other hand, this study focussed only on White Finnish males without detailed information regarding their genetic risk for CHD, which limits possibilities to generalise its findings [57]. Moreover, this study refers specifically to CHD, ICD 10 codes I20 − I25, as an endpoint, and because the main CHD risk factors are to some extent outcome sensitive [26], i.e. explanatory powers and the order of importance among risk factors slightly differ across endpoints, such as CHD, an acute myocardial infarction (AMI), and a fatal AMI, the present results are not directly generalisable to AMI and sudden cardiac death, although them are typical manifestations of CHD. Our previous paper provides analyses to verify this outcome sensitivity [26]. To control possible bias originated from inaccuracies related to exclusions of study participants based on the ergometer test we executed statistical analyses and reported their results also concerning the dataset without exclusions. The exclusions did not affect the results of this study, which is in accordance with our previous findings [26].

## Conclusions

Considering interactions across the main CHD risk factors together with their time dependencies in a survival model on CHD incidence changed the order of importance among the risk factors compared to a simple multiplicative model. Without considering both interactions and time dependence the survival model stressed glycemic status as the strongest modifiable risk factor, whereas the extended model emphasised S-LDL-C status that research, traditionally, has identified as a pivotal CHD risk factor. Age overcame the family history of CHD as the strongest non-modifiable risk factor. This study seriously reminds the benefits of dealing simultaneously with multiplicative, additive, and interactive associations for validity.

From the clinical perspective, this study suggests high S-LDL-C concentrations being a very strong explanator of CHD risk among men younger than 50. Also, obesity is a strong explanator, but both age and smoking confound the relationship between CHD and obesity. Furthermore, this study proposes the order of importance among the main CHD risk factors, also in the clinical context, may slightly differ from the conventional order. Smoking, for example, could be a so strong predictor of non-CHD mortality that its role in the development of CHD, at least, at the population level may show moderate.

## Supplementary Material

Supplemental Material

## Data Availability

On request, the University of Eastern Finland’s Institute of Public Health and Clinical Nutrition can admit an access to the KIHD database.
