# Supplementary material for: Epidemiological analysis of coronary heart disease and its main risk factors: are their associations multiplicative, additive, or interactive?
Source: Ann Med. 2022 May 23;54(1):1500–10. doi: 10.1080/07853890.2022.2078875 (PMC9132387; doi:10.1080/07853890.2022.2078875)
Supplement: Supplemental Material [file IANN_A_2078875_SM7343.pdf]

Supplementary table 1. 95% CIs for risk (RR) to detect multiplicative interaction and the interaction contrast ratio (ICR) together with the risk difference (RD) to detect additive interaction.

Interaction	Multiplicative[1]	Multiplicative[2]	Additive[1]	Additive[2]
Age ×<br>No family history (0)<br>Family history (1)	$p = 0.640$<br>RR0: 1.56–2.60<br>RR1: 1.15–1.69	$p = 0.228$<br>RR0: 1.63–2.51<br>RR1: 1.21–1.64	ICR: -1.27–0.85<br>RD0: 0.13–0.24<br>RD1: 0.06–0.20	ICR: -1.23–1.91<br>RD0: 0.16–0.27<br>RD1: 0.09–0.21
Diabetes ×<br>No family history (0)<br>Family history (1)	$p = 0.969$<br>RR0: 1.16–1.92<br>RR1: 1.14–1.74	$p = 0.671$<br>RR0: 1.26–1.81<br>RR1: 1.15–1.53	ICR: -1.02–2.43<br>RD0: 0.05–0.26<br>RD1: 0.05–0.28	ICR: -1.29–3.43<br>RD0: 0.09–0.27<br>RD1: 0.07–0.24
Smoking ×<br>No family history (0)<br>Family history (1)	$p = 0.127$<br>RR0: 0.79–1.16<br>RR1: 0.98–1.35	$p = 0.131$<br>RR0: 0.83–1.12<br>RR1: 0.99–1.25	ICR: -1.28–0.05<br>RD0: -0.07–0.05<br>RD1: -0.01–0.13	ICR: -0.12–2.34<br>RD0: -0.07–0.04<br>RD1: 0.00–0.11
Obesity ×<br>No family history (0)<br>Family history (1)	$p = 0.594$<br>RR0: 0.97–1.49<br>RR1: 1.06–1.51	$p = 0.825$<br>RR0: 0.94–1.34<br>RR1: 1.01–1.31	ICR: -0.39–1.41<br>RD0: -0.01–0.14<br>RD1: 0.02–0.19	ICR: -0.95–1.68<br>RD0: -0.02–0.11<br>RD1: 0.00–0.14
High S-LDL-C ×<br>No family history (0)<br>Family history (1)	$p = 0.665$<br>RR0: 1.20–1.70<br>RR1: 1.22–1.66	$p = 0.809$<br>RR0: 1.20–1.58<br>RR1: 1.19–1.48	ICR: -0.16–1.31<br>RD0: 0.06–0.17<br>RD1: 0.08–0.22	ICR: -0.24–2.32<br>RD0: 0.07–0.17<br>RD1: 0.09–0.19
Hypertension ×<br>No family history (0)<br>Family history (1)	$p = 0.291$<br>RR0: 1.23–1.78<br>RR1: 1.05–1.46	$p = 0.351$<br>RR0: 1.40–1.92<br>RR1: 1.19–1.52	ICR: -0.78–0.62<br>RD0: 0.07–0.18<br>RD1: 0.02–0.15	ICR: -0.84–1.69<br>RD0: 0.12–0.22<br>RD1: 0.08–0.19
Diabetes ×<br>Age <50 (0)<br>Age >50 (1)	$p = 0.563$<br>RR0: 1.14–2.60<br>RR1: 1.13–1.61	$p = 0.813$<br>RR0: 1.01–2.08<br>RR1: 1.20–1.51	ICR: -2.08–2.33<br>RD0: 0.02–0.34<br>RD1: 0.05–0.23	ICR: -0.50–2.83<br>RD0: 0.00–0.27<br>RD1: 0.09–0.23
Smoking ×<br>Age <50 (0)<br>Age >50 (1)	$p = 0.767$<br>RR0: 0.76–1.38<br>RR1: 0.93–1.22	$p = 0.898$<br>RR0: 0.83–1.35<br>RR1: 0.96–1.16	ICR: -0.55–0.81<br>RD0: -0.07–0.08<br>RD1: -0.03–0.08	ICR: -0.49–0.71<br>RD0: -0.05–0.09<br>RD1: -0.02–0.07
Obesity ×<br>Age <50 (0)<br>Age >50 (1)	$p = 0.032$<br>RR0: 1.27–2.33<br>RR1: 0.95–1.30	$p = 0.174$<br>RR0: 1.05–1.82<br>RR1: 0.96–1.20	ICR: -2.22–0.42<br>RD0: 0.06–0.27<br>RD1: -0.02–0.11	ICR: -1.30–0.59<br>RD0: 0.01–0.21<br>RD1: -0.02–0.09
High S-LDL-C ×<br>Age <50 (0)<br>Age >50 (1)	$p = 0.002$<br>RR0: 1.59–2.79<br>RR1: 1.07–1.38	$p = 0.005$<br>RR0: 1.48–2.34<br>RR1: 1.10–1.32	ICR: -2.24–0.29<br>RD0: 0.12–0.28<br>RD1: 0.03–0.13	ICR: -1.45–0.52<br>RD0: 0.12–0.27<br>RD1: 0.04–0.13
Hypertension ×<br>Age <50 (0)<br>Age >50 (1)	$p = 0.932$<br>RR0: 1.04–1.84<br>RR1: 1.14–1.50	$p = 0.194$<br>RR0: 1.07–1.73<br>RR1: 1.31–1.62	ICR: -0.27–1.27<br>RD0: 0.01–0.15<br>RD1: 0.06–0.16	ICR: 0.60–1.92<br>RD0: 0.02–0.16<br>RD1: 0.12–0.21
Smoking ×<br>No diabetes (0)<br>Diabetes (1)	$p = 0.956$<br>RR0: 0.92–1.20<br>RR1: 0.74–1.43	$p = 0.194$<br>RR0: 0.97–1.18<br>RR1: 0.72–1.16	ICR: -1.26–1.75<br>RD0: -0.03–0.06<br>RD1: -0.15–0.18	ICR: -1.55–0.61<br>RD0: -0.01–0.07<br>RD1: -0.18–0.08
Obesity ×<br>No diabetes (0)<br>Diabetes (1)	$p = 0.923$<br>RR0: 1.01–1.38<br>RR1: 0.82–1.51	$p = 0.980$<br>RR0: 0.96–1.23<br>RR1: 0.86–1.30	ICR: -1.22–1.92<br>RD0: 0.00–0.13<br>RD1: -0.10–0.21	ICR: -0.95–1.35<br>RD0: -0.02–0.09<br>RD1: -0.09–0.15

[1]Study participants free of coronary heart disease at baseline ($n = 1958$). [2]All study participants ($n = 2682$). Frames highlight statistically significant interactions.

For Peer Review Only

Supplementary table 1. Continues.

Interaction	Multiplicative[1]	Multiplicative[2]	Additive[1]	Additive2
High S-LDL-C ×<br>No diabetes (0)<br>Diabetes (1)	*p* = 0.478<br>RR0: 1.28–1.65<br>RR1: 0.87–1.60	*p* = 0.542<br>RR0: 1.26–1.52<br>RR1: 0.95–1.44	ICR: -1.59–2.25<br>RD0: 0.09–0.18<br>RD1: -0.07–0.24	ICR: -1.08–1.95<br>RD0: 0.10–0.17<br>RD1: -0.03–0.21
Hypertension ×<br>No diabetes (0)<br>Diabetes (1)	*p* = 0.755<br>RR0: 1.19–1.54<br>RR1: 0.82–1.73	*p* = 0.812<br>RR0: 1.32–1.62<br>RR1: 1.01–1.93	ICR: -1.68–1.77<br>RD0: -0.09–0.25<br>RD1: 0.06–0.15	ICR: -0.66–2.14<br>RD0: 0.11–0.19<br>RD1: 0.02–0.32
Obesity ×<br>No smoking (0)<br>Smoking (1)	*p* = 0.034<br>RR0: 1.15–1.57<br>RR1: 0.66–1.28	*p* = 0.007<br>RR0: 1.11–1.41<br>RR1: 0.69–1.12	ICR: -1.50–(-0.01)<br>RD0: 0.05–0.18<br>RD1: -0.04–0.09	ICR: -1.29–(-0.19)<br>RD0: 0.04–0.16<br>RD1: -0.15–0.04
High S-LDL-C ×<br>No smoking (0)<br>Smoking (1)	*p* = 0.830<br>RR0: 1.22–1.62<br>RR1: 1.17–1.77	*p* = 0.953<br>RR0: 1.22–1.51<br>RR1: 1.15–1.56	ICR: -0.52–0.68<br>RD0: 0.07–0.18<br>RD1: 0.06–0.21	ICR: -0.49–0.50<br>RD0: 0.08–0.18<br>RD1: 0.06–0.19
Hypertension ×<br>No smoking (0)<br>Smoking (1)	*p* = 0.649<br>RR0: 1.16–1.58<br>RR1: 1.15–1.75	*p* = 0.887<br>RR0: 1.34–1.73<br>RR1: 1.25–1.72	ICR: -0.39–0.78<br>RD0: 0.05–0.16<br>RD1: 0.05–0.21	ICR: -0.47–0.68<br>RD0: 0.12–0.21<br>RD1: 0.10–0.23
High S-LDL-C ×<br>No obesity (0)<br>Obesity (1)	*p* = 0.870<br>RR0: 1.25–1.63<br>RR1: 1.09–1.76	*p* = 0.895<br>RR0: 1.24–1.51<br>RR1: 1.08–1.56	ICR: -0.53–1.67<br>RD0: 0.08–0.18<br>RD1: 0.04–0.26	ICR: -0.54–1.05<br>RD0: 0.09–0.17<br>RD1: 0.04–0.22
Hypertension ×<br>No obesity (0)<br>Obesity (1)	*p* = 0.953<br>RR0: 1.17–1.54<br>RR1: 0.91–1.87	*p* = 0.676<br>RR0: 1.33–1.64<br>RR1: 1.13–2.18	ICR: -0.91–0.87<br>RD0: 0.06–0.15<br>RD1: -0.03–0.23	ICR: -1.55–0.25<br>RD0: 0.12–0.20<br>RD1: 0.07–0.29
Hypertension ×<br>No high S-LDL-C (0)<br>High S-LDL-C (1)	*p* = 0.100<br>RR0: 1.31–1.91<br>RR1: 1.03–1.42	*p* = 0.074<br>RR0: 1.47–2.01<br>RR1: 1.16–1.50	ICR: -1.00–0.69<br>RD0: 0.08–0.19<br>RD1: 0.01–0.15	ICR: -0.65–0.88<br>RD0: 0.14–0.24<br>RD1: 0.07–0.19

[1]Study participants free of coronary heart disease at baseline (n = 1958). [2]All study participants (n = 2682).
Frames highlight statistically significant interactions.

For Peer Review Only
